# Therapeutic Effect of Intestinal Autochthonous *Lactobacillus reuteri* P16 Against Waterborne Lead Toxicity in *Cyprinus carpio*

**DOI:** 10.3389/fimmu.2018.01824

**Published:** 2018-08-07

**Authors:** Sib Sankar Giri, Saekil Yun, Jin Woo Jun, Hyoun Joong Kim, Sang Guen Kim, Jeong Woo Kang, Sang Wha Kim, Se Jin Han, V. Sukumaran, Se Chang Park

**Affiliations:** ^1^Laboratory of Aquatic Biomedicine, College of Veterinary Medicine, Research Institute for Veterinary Science, Seoul National University, Seoul, South Korea; ^2^Department of Biotechnology, Periyar Maniammai University, Thanjavur, India

**Keywords:** probiotics, lactic acid bacteria, Pb, common carp, oxidative stress, immune parameters, gene expression

## Abstract

Harmful effects of heavy metals are myriad. Lead (Pb) from soil and atmosphere contaminates water bodies and affects the aquatic animals. Our previous study confirmed the *in vitro* probiotic potential of *Lactobacillus reuteri* against Pb toxicity, but further investigation is necessary for gaining insights into the related protection mode. Therefore, in this study, we investigated the protective effects of the potential probiotic *L. reuteri* P16 against waterborne Pb exposure-induced toxicity in the freshwater fish *Cyprinus carpio*. Fish (average weight: 23.16 ± 0.73 g) were allocated to four groups (control, Pb only, Pb + *L. reuteri* P16, and *L. reuteri* P16 only) and Pb groups were exposed to waterborne Pb (1 mg L^−1^) for 6 weeks. *L. reuteri* P16 (10^8^ CFU g^−1^) supplemented diet was provided twice daily. Growth performances, hemato-biochemical parameters, innate immune responses, intestinal microbiota, and Pb accumulation in tissues were measured at the end of the trial. When the fish were exposed to Pb, dietary supplementation of *L. reuteri* P16 effectively decreased mortality and accumulation of Pb in tissues, and improved the growth performance. Co-treatment with Pb and *L. reuteri* P16 alleviated Pb exposure-induced oxidative stress, reversed alterations in hemato-biochemical parameters, improved innate immune parameters, and restored intestinal enzymatic activities. Moreover, *L. reuteri* P16 supplementation reversed the changes in intestinal microbiota in Pb-exposed fish. Furthermore, Pb exposure decreased the expressions of pro-inflammatory cytokines (TNF-α, IL-1β). However, the expression of heat shock proteins (HSP70 and HSP90) increased, which might have increased the cellular stress. Interestingly, the Pb-induced alterations of gene expressions were reversed by *L. reuteri* P16 supplementation. Thus, dietary administration of the potential probiotic *L. reuteri* P16 had several beneficial effects on growth performance and immune responses, decreased Pb accumulation in tissues, and reversed alterations in hematological responses of *C. carpio*. Furthermore, it offered direct protection against Pb-induced oxidative stress. Therefore, *L. reuteri* P16 may be a novel dietary supplement for enhancing growth performance and preventing Pb-exposure-induced toxicity in fish in aquaculture and aquatic products.

## Introduction

In recent years, contamination of aquatic environments by heavy metals is increasing rapidly. These heavy metals have caused lot of problems due to their toxicity, and it poses threat to both aquaculture and food safety (1). The natural aquatic systems are being extensively contaminated with heavy metals released from industrial and other anthropogenic activities. Among the various heavy metals, lead (Pb) is one of the most toxic and occupies the second place in Priority List of Hazardous Substances (2). Due to higher levels in the food web, fish can accumulate many metals, and the accumulation patterns in fish rely on uptake and elimination rates (3). Pb accumulation in fish tissues depends on the type of metal, exposure duration, and concentration, salinity, temperature, water quality, fish species, and metabolic activity of fish (4). Pb is mutagenic and teratogenic in humans and animals (5). Prolonged Pb exposure can produce adverse effects on pituitary function, gonadosomatic index, and oocyte growth, chromosomal aberrations, and causes neurological disorders, DNA damage, and scoliosis in freshwater fish (6–8). Furthermore, Pb showed deleterious effects on gut-associated lymphoid tissues of *Channa punctatus* (8). In addition to economic loss to aquaculture, heavy metal contamination in aquaculture poses great risks to human health because it can accumulate and enter the food chain (9). Therefore, eco-friendly and affordable strategies of controlling heavy metal (Pb) contamination in aquaculture is increasing in importance.

Until date, no treatment method for chronic Pb poisoning has been developed. Although chelation therapy has been used to promote heavy metal excretion, chelators for Pb toxicity have been reported to be deficient in terms of safety and efficacy (10). Gut microbiota of fish play important roles in metabolism, immunity, and pathogen resistance (11). Allochthonous (transient) and autochthonous (adherent) microbes normally reside in gastrointestinal (GI) tract of fish, and the latter type has been well investigated for their numerous beneficial effects on the host (11). Colonization of the GI tract of fish larvae completes within few hours after hatching. The colonization by normal or protective microbes prevents colonization by potential invaders and maintains the overall health of fish (12). Lactic acid bacteria (LAB) are classified as “generally regarded as safe” and have been widely used in aquaculture (13). LAB with potential heavy metal adsorption capacity have been isolated and identified earlier (14–16). They remove heavy metals *in vivo* and *in vitro* (14, 17, 18). Recently, Yu et al. (13) demonstrated that dietary *Lactobacillus plantarum* could alleviates the waterborne aluminum toxicity in tilapia. In another study, Zhai et al. (14) demonstrated that dietary probiotic *L. plantarum* CCFM8610 has potential to prevent cadmium-exposure induced problems in tilapia aquaculture. Therefore, LAB could be exploited further for their possible role in alleviating heavy metal toxicity in aquaculture.

Immunostimulants and other biological factors, such as probiotics, can trigger the defense system, even under stressful conditions, thereby reducing the deleterious effects caused by various biological, chemical, and physiological stresses (19). Until date, numerous studies have demonstrated that probiotics can stimulate immunity in teleosts both under *in vivo* and *in vitro* conditions. Available literature indicates that several probiotics either individually or in combination can enhance both systemic and local immunity in fish (20, 21). Probiotic activities of LAB and other bacteria isolated from the GI tract of fish have been demonstrated in several studies (22–25). However, protective effect of those autochthonous probiotic bacteria against heavy metal-induced toxicity in fish has not been reported yet. Various microorganisms have been studied and strategically used for bioremediation of water polluted with heavy metals (26, 27). Recently, it has been found that LAB, such as *L. plantarum, Lactobacillus rhamnosus, Lactobacillus reuteri*, and *Bifidobacterium lactis* can bind and remove heavy metals *in vitro* from water and environment (15, 27). Yi et al. (17) demonstrated that *Leuconostoc mesenteroides* isolated from fermented kimchi has strong lead resistance and removal capacity. It has been reported that dietary supplementation of *L. plantarum* CCFM8611 (at 10^8^ CFU g^−1^) isolated from environmental samples alleviated Pb-induced oxidative stress, reversed digestive enzyme activities and innate immune status, and ameliorated the growth performance in Pb-induced fish (18). Hematological parameters are reliable indicators of physiological status of aquatic animals under stress due to metal exposure.

Recently, we have isolated Pb-resistant LAB from the gut content of carp *Cyprinus carpio* (28). Among those isolates, *L. reuteri* P16 was found to exhibit strong Pb-binding and tolerance abilities. *L. reuteri* P16 exhibited various probiotic properties, such as a high level of tolerance to both acid (pH 2.0 and 3.0) and bile (0.3% Oxgall) exposure, good adhesion to intestinal mucosa, strong inhibition of fish-pathogen growth *in vitro*, and susceptibility to several clinically effective antibiotics (28). Furthermore, “intact cells” and cell-free supernatants of P16 had stronger antioxidant abilities. Those results indicated that it is important to investigate efficacy of *L. reuteri* P16 in providing protection against toxicity caused by Pb-exposure in aquaculture. *C. carpio* is an important aquaculture species worldwide. It is the third most widely cultivated and commercially important freshwater fish species in the world. In addition, it is recognized as a good biological model because it is easy to handle, culture, and maintain in the laboratory. Therefore, this study was aimed to investigate the effect of dietary supplementation of intestinal autochthonous *L. reuteri* P16 on the growth performance, hematological, and blood biochemical parameters, and intestinal enzymatic activities of the carp *C. carpio* exposed to waterborne Pb. In addition, effects of P16 on cytokine gene expression, and Pb accumulation in tissues of carp were investigated.

## Materials and Methods

### Potential Probiotic Strain

*Lactobacillus reuteri* P16 isolated from the gut content of *C. carpio*, exhibited *in vitro* probiotic properties, and excellent Pb-binding and Pb-tolerance capacities (28). This strain was cultured in de Man, Rosaga, and Sharpe (MRS) broth for 24 h at 37°C.

### Preparation of Diet for Fish

The basal diet containing 28.8% protein, 7.16% lipid, and 12.36% ash was prepared as described in a previous study (29). The culture broth was centrifuged at 8,000 × *g* for 15 min at 4°C to precipitate the cells. The initial bacterial concentration (1.32 × 10^9^ cells mL^−1^) was diluted to the desired concentration using the phosphate buffer saline (pH 7.4). P16 was added to the basal diet at a final concentration of 1 × 10^8^ CFU g^−1^ (approximately). Based on a preliminary study, P16 concentration was chosen as 1 × 10^8^ CFU g^−1^ (unpublished data). The number of viable bacterial cells in the diet was determined *via* plate counting on MRS agar plates. The ingredients were blended thoroughly into a mixture that and then pelleted, air-dried, ground, and sieved into appropriate pellet sizes. The experimental feed was prepared weekly and stored at 4°C.

### Experimental Design

*Cyprinus carpio* (average body length: 11.2 ± 0.8 cm; average weight: 23.16 ± 0.73 g; approximate age: 105 days) obtained from a local fish farm were acclimatized to laboratory conditions in 500 L quarantine tanks for 2 weeks at 24 ± 2°C and fed a basal diet (29). Approximately 20% of the water in all the tanks was replaced daily, and 100% of the water was replaced weekly. Basic physicochemical parameters of the water were monitored weekly; oxygen and ammonia concentrations were 6.1–7.3 and 0.03–0.06 mg L^–1^, respectively, and pH ranged from 7.0 to 7.6. This study was conducted in accordance with the “Guidelines on the Regulation of Scientific Experiments on Animals,” CPCSEA (http://cpcsea.nic.in), Govt. of India and the experimental protocols were approved by the bioethical committee of the Periyar Maniammai University (PMU/Biotech/10.01.2009).

In total, 180 fish were randomly distributed into the four experimental groups. In each independent experimental group, 45 fish were divided into three tanks (15 fish per tank), and each tank held 200 L water. The following groups were maintained:

I. Control group: the fishes were fed with basal diet and kept in Pb-free water.
II. Pb-only group: the fishes were exposed to waterborne Pb (1 mg L^−1^) and fed with the basal diet.
III. Pb + P16 group: the fishes were exposed to waterborne Pb and fed on diet supplemented with *L. reuteri* P16.
IV. P16 group: the fishes were fed on diet containing 10^8^ CFU g^−1^ of *L. reuteri* P16, and kept in Pb-free water.

The experimental feeding period selected was 6 weeks. The sublethal Pb dose (1 mg L^−1^) was chosen based on earlier reports (14, 18). The fishes were fed experimental diets at 3−5% of body weight two times daily (09:00 and 18:00 h). For Pb-treated groups, water was exchanged with Pb-containing water every 2 days to maintain a constant Pb level. For other groups, 20% of the water was exchanged daily, and 100% of the water was exchanged once in a week. Water samples were collected daily for Pb level determination using a flame atomic absorption spectrophotometer.

### Growth Performance and Sample Collection

The fishes were weighed at the beginning and end of the 6-week feeding trial. Growth performance [i.e., percent weight gain (PWG; grams per fish), specific growth rate (SGR), and feed conversion ratio (FCR)] and survival rates of fish were calculated using the following formula:

$$\mathrm{PWG}=(W_{t}-W_{0}/W_{0})\times 100\%$$

$$\mathrm{SGR}=[(\text{ln }W_{t}-\text{ln }W_{0})/t]\times 100\%$$

$$\mathrm{FCR}=\mathrm{FI}/(W_{t}-W_{0})$$

where, *W_t_* and *W*_0_ are the final and initial weight of the fishes, respectively, *t* is the duration of feeding (in days); and FI is the feed intake.

At the end of the feeding trial, the fish were starved for 24 h before sampling. Ten fish were collected from each tank (i.e., 10 fish × 3 tanks = 30 fish per group) and euthanized with 200 mg L^−1^ of MS-222 (Ethyl 3-aminobenzoate methanesulfonate; Sigma-Aldrich, St. Louis, MO, USA). Immediately, blood samples were collected from the caudal vein using a 2-mL syringe. Pooled blood samples were divided into two aliquots: one was centrifuged (4,000 × *g*, 10 min, 4°C) to collect serum and the other was stored in anti-coagulative tubes (EDTA-2K).

Tissue samples from gills, spleen, liver, brain, and kidney were collected from those fishes and stored in metal-free Eppendorf tubes at −20°C. Furthermore, intestinal samples from nine fish per group (3 fish × 3 tanks = 9 fish) were used for the quantification of intestinal microbiota.

### Hematological Parameters

Hematological parameters, including white blood cell (WBC) count, red blood cell (RBC) count, hematocrit value (Hct, %), and hemoglobin (Hb) level, were measured using standard techniques. Serum total protein was estimated using a commercial kit (Abcam, India). Cholesterol level was measured calorimetrically using a commercial kit (Sigma-Aldrich, USA).

### Blood Biochemical Parameters

Malondialdehyde (MDA) content was measured *via* barbituric acid reaction chronometry (30). Total myeloperoxidase (MPO) content in blood serum was measured using a commercial kit (Abcam, USA). Serum aspartate aminotransferase (AST), alanine aminotransferase (ALT), and alkaline phosphatase (ALP) activities were determined using the Hitachi 912 clinical chemistry automatic analyzer. Superoxide dismutase (SOD) activity was determined with an enzymatic assay method using a commercial kit (Randox, Crumlin, UK). Blood creatinine level was measured using commercial assay kit (Sigma-Aldrich, USA). Glutathione peroxidase (GPx) was measured with an enzymatic assay using commercial kit (Abcam, USA). Serum lysozyme activity was measured with commercial assay kit (Sigma-Aldrich). Leukocyte phagocytic activity was measured according to the method described by Dotta et al. (31).

### Intestinal Enzymatic Activities and Quantification of Intestinal Microbiotia

For this experiment, whole intestines from six fish of each group were sampled aseptically and rinsed with distilled water. Intestines were homogenized and the activity of digestive enzymes was determined. Amylase and protease activities were measured as described by Pavasovic et al. (32), and lipase activity was evaluated by using a commercial kit (Sigma-Aldrich).

The entire intestinal tract was removed aseptically (*n* = 9) from the sampled fish, washed thoroughly with sterile saline (0.85% NaCl), and homogenized (Potter–Elvehjem Tissue Homogenizer, NW Kennesaw, GA, USA) to isolate the intestinal microbiological communities (33). The homogenate was serially diluted to 10^−7^ with sterile saline. Dilutions (100 µL) were spread in triplicate onto plate count agar (PCA) and MRS agar plates. MRS cultures were incubated anaerobically (5% CO_2_) at 37°C for 72 h, while PCA cultures were incubated at 37°C for 24–72 h, aerobically, for determining the total count. The viability was recorded as colony-forming units (CFU) per milliliter and cell concentration was expressed as log (CFU mL^−1^).

### Determination of Pb Level in Tissues

**Figure 1:**
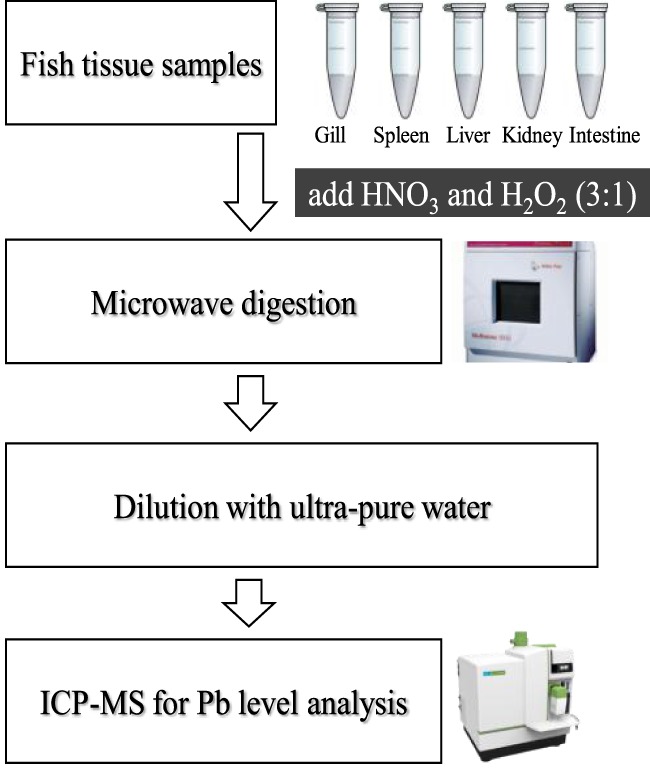
Transfer system of tissue samples for the determination of the Pb level.

Tissue samples were transferred to metal-free vessels and digested in concentrated HNO_3_ and H_2_O_2_ (3:1; v/v) using a microwave digestion system (Microwave 3000; Anton Paar GmbH, Austria) (14). The Pb concentrations in all the tissue samples were determined using atomic absorption spectrophotometer (PerkinElmer, USA) (Figure 1).

### Immune Gene Expression Analysis

Head-kidney were dissected from six fish per group after they were anesthetized with MS-222, and total RNA was isolated from those tissues using TRIZOL reagent (Invitrogen, USA) according to the manufacturer’s instructions. Real-time polymerase chain reaction analysis of IL-1β, TNF-α, HSP70, HSP90, and the housekeeping gene β-actin were carried out following standard protocols (29). The primer sequences and thermo cycling conditions are shown in Table 1. After amplification, melting curve analysis was performed to verify the accuracy of each amplicon. All the samples were run in parallel with the β-actin to normalize cDNA loading. Gene expression results were analyzed using the 2^−ΔΔCT^ method after verification that the primers amplified with an efficiency of approximately 100% (34).

**Table 1:** Real-time primer sequences and thermocycling conditions.

Target gene	Primer sequence (5′–3′)	Thermocycling conditions	Reference/accession no.
TNF-α	CTCAACAAGTCTCAGAACAATCAGG TCCTGGTTCCTTCTCCAATCTAGCT	95°C 30 s, 40 cycles of 95°C 5 s, 61.1°C 30 s, and 72°C 30 s	(19)

IL-1β	ATCTTGGAGAATGTGATCGAAGAG GATACGTTTTTGATCCTCAAGTGTGAAG	95°C 30 s, 40 cycles of 95°C 5 s, 61.5°C 30 s, and 72°C 30 s	(19)

HSP70	GGC AGA AAG TTT GAT GAC CCA GCA ATC TCC TTC ATA TTC ACC	95°C 30 s, 40 cycles of 95°C 5 s, 61.1°C 30 s, and 72°C 30 s	(15)

HSP90	GGAAATCTTCCTCCGAGAGC CCGAATTGACCGATCATAGA	95°C 30 s, 40 cycles of 95°C 5 s, 61.1°C 30 s, and 72°C 30 s	(28)

β-actin	GACTTCGAGCAGGAGATGG CAAGAAGGATGGCTGGAACA	95°C 30 s, 40 cycles of 95°C 5 s, 62.4°C 30 s, and 72°C 30 s	(19)

### Statistical Analyses

Independent data were obtained and statistical significance testing were performed. The homogeneity of variance was checked by analysis of variance, and chi-square test was used to analyze the data. Multiple comparisons were performed with Tukey’s test to analyze the differences between independent experimental groups and treatments. All statistical analyses were performed using the OriginPro software (version 8; OriginLab Corporation, Northampton, MA, USA). Significance level was set at *P* < 0.05.

## Results

### Growth Performance

**Figure 2:**
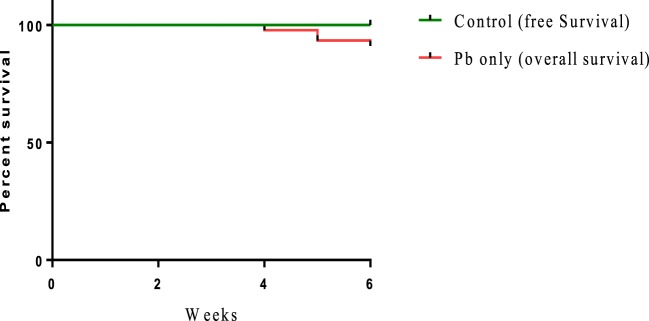
Kaplan–Meier survival curve [cumulative survival (%) over time (weeks)] of *Cyprinus carpio* exposed waterborne Pb.

Sublethal exposure to waterborne Pb resulted in the inhibition of growth performance and increased the mortality of *C. carpio* (Table 2). Survival in the Pb-exposed group was 91.1%, but that in other groups was 100% and this shown by plotting Kaplan–Meier survival curve (Figure 2). Typical symptoms include spinal deformity and blackening of the caudal region were observed in fish exposed to Pb only. Final weight gain (FWG), PWG, and SGR were significantly lowered in the Pb-only group than in the control group or the group fed with *L. reuteri* P16 alone. The FCR value was significantly higher in Pb-only group, but dietary supplementation with *L. reuteri* P16 decreased the FCR value, and it was the lowest in the group fed with *L. reuteri* P16 only. Supplementation with *L. reuteri* P16 prevented death caused by Pb exposure, as revealed by 100% survival in the Pb + P16 group. Growth performance (FWG, PWG, and SGR) in the Pb + P16 group was not significantly higher than that of the Pb-only group. However, administration of *L. reuteri* P16 alone significantly increased the FG, PWG, and SGR of the fishes, when compared with the other groups. FCR was the lowest in the P16 group (Table 2).

**Table 2:** Effect of dietary supplementation on growth performance of *Cyprinus carpio* during 6-week trial.

Group	Initial weight (g)	Final weight (g)	PWG (%)	SGR (%)	FCR	Survival (%)
Control	23.26 ± 0.38^a^	32.86 ± 1.07^a^	37.61 ± 1.26^a^	0.90 ± 0.2^a^	2.04 ± 0.08^a^	100
Pb only	23.21 ± 0.71^a^	29.73 ± 1.14^b^	30.11 ± 0.82^b^	0.71 ± 0.03^b^	2.47 ± 0.13^b^	91.1
Pb + P16	23.14 ± 0.63^a^	30.91 ± 1.52^b^	33.26 ± 0.64^b^	0.83 ± 0.07^b^	2.28 ± 0.13^c^	100
P16 only	23.18 ± 0.52^a^	36.0.8 ± 0.96^c^	54.76 ± 1.77^c^	1.22 ± 0.6^c^	1.84 ± 0.6^d^	100

*PWG, percent weight gain; SGR, specific growth rate; FCR, feed conversion ratio*.

*Values in the same column with different superscript small letters are significantly different (*P* < 0.05)*.

*Values are presented as mean ± SD (*n* = 45 fish in each group)*.

### Hematological Parameters

Pb exposure significantly decreased the RBC, WBC, Hct, Hb, and total protein level in blood, when compared with those in the control (Table 3). However, decreases of those parameters were reversed by *L. reuteri* P16 supplementation. Levels of RBC, WBC, Hb, Hct, and total protein recovered in the Pb + P16 group, and the differences with the control group were not significant. Furthermore, blood cholesterol level, which increased significantly under Pb-exposure stress, was recovered by probiotic feeding (Table 3). However, supplementation with *L. reuteri* P16 alone had no significant effect on these parameters, except total protein level, which was significantly increased by probiotic supplementation.

**Table 3:** Effect of *Lactobacillus reuteri* P16 supplementation on the hematological parameters of *Cyprinus carpio*.

Group	Parameters					
	RBC (×10^6^ mm^**−**3^)	WBC (×10^3^ mm^**−**3^)	Hct (%)	Hb (g dL^**−**1^)	Total protein (g dL^**−**1^)	Cholesterol (mg dL^**−**1^)
Control	1.69 ± 0.07^ac^	4.21 ± 0.13^a^	24.03 ± 0.83^a^	5.37 ± 0.13^a^	2.87 ± 0.04^a^	58.06 ± 2.8^a^
Pb only	1.53 ± 0.08^b^	3.48 ± 0.09^b^	19.86 ± 1.07^b^	3.89 ± 0.11^b^	2.41 ± 0.07^b^	63.57 ± 2.4^b^
Pb + P16	1.58 ± 0.05^ba^	3.96 ± 0.14^ab^	21.92 ± 0.74^b^	4.92 ± 0.08^a^	2.78 ± 0.06^ba^	61.19 ± 3.3^ba^
P16 only	1.76 ± 0.09^c^	4.54 ± 0.11^a^	25.13 ± 1.28^a^	6.08 ± 0.14^c^	3.62 ± 0.11^c^	57.88 ± 3.02^a^

*RBC, red blood cell; WBC, white blood cell; Hct, hematocrit; Hb, hemoglobin*.

*Data are presented as mean ± SD (*n* = 30 fish from each group). Means in the same column with different superscript small letters are significantly different (*P* < 0.05)*.

### Blood Biochemical Parameters

Among biochemical parameters, levels of AST (163.1 ± 4.7 U mL^−1^), ALT (68.41 ± 2.12 U mL^−1^), and creatinine (0.37 ± 0.07 U mL^−1^) increased significantly in (*P* < 0.05) in Pb-only group, but these alterations were reversed to the normal level in Pb + P16 group (Table 4). However, feeding with *L. reuteri* P16 alone had no significant effect on those parameters, except AST level, which decreased significantly in this group, as compared with that in the control. Furthermore, serum ALP and MPO levels, which decreased significantly in Pb-only group, were recovered in the Pb + P16 group, but these levels were slightly lower than those in the control group. However, *L. reuteri* P16 treatment alone significantly increased the MPO level than that in the control or other groups. In case of MPO level, *L. reuteri* P16 treatment alone had no significant effect on it.

**Table 4:** Effect of *Lactobacillus reuteri* P16 supplementation on the blood biochemical parameters of *Cyprinus carpio*.

Group	Parameters				
	ALP (IU L^**−**1^)	AST (U mL^**−**1^)	ALT (U mL^**−**1^)	MPO (U L^**−**1^)	Creatinine (mg dL^**−**1^)
Control	21.38 ± 1.26^a^	104.3 ± 3.4^a^	32.06 ± 1.46^a^	34.68 ± 1.83^a^	0.26 ± 0.08^ac^
Pb only	17.82 ± 1.14^b^	163.1 ± 4.7^b^	68.41 ± 2.12^b^	29.57 ± 1.24^b^	0.37 ± 0.07^b^
Pb + P16	20.87 ± 0.83^a^	107.6 ± 2.8^a^	39.6 ± 1.38^c^	32.92 ± 1.67^a^	0.29 ± 0.08^a^
P16 only	22.04 ± 1.06^a^	98.9 ± 3.1^c^	31.33 ± 1.6^a^	37.14 ± 0.92^c^	0.24 ± 0.03^c^

*ALP, alkaline phosphatase; AST, aspartate aminotransferase; ALT, alanine aminotransferase; MPO, myeloperoxidase*.

*Values in the same column with different superscript small letters are significantly different (*P* < 0.05)*.

*Values are presented as mean ± SD (*n* = 30)*.

### Serum Oxidative Parameters

**Figure 3:**
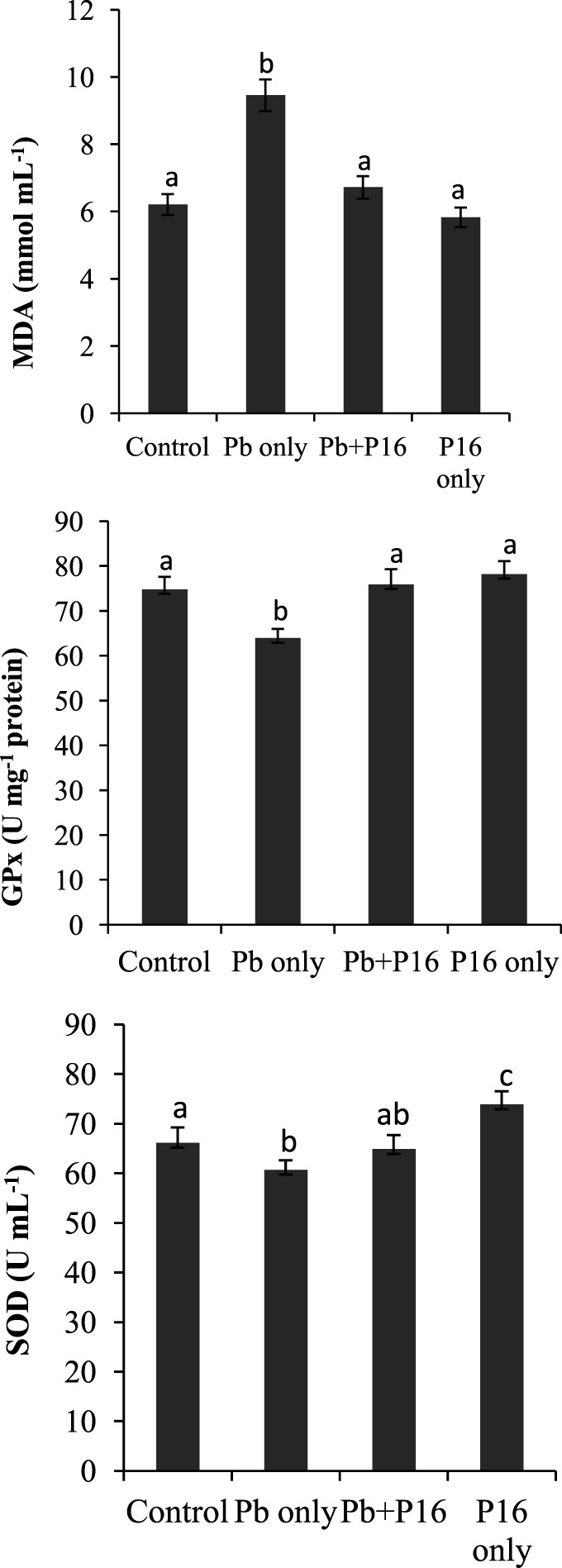
Influence of *Lactobacillus reuteri* P16 supplementation on the oxidative parameters in the blood of *Cyprinus carpio*. Significant differences between groups are indicated with different superscript letters. Results are presented as mean ± SEM (*n* = 30).

Malondialdehyde level was increased significantly (*P* < 0.05) in the Pb-only group, but *L. reuteri* P16 treatment reversed the MDA level to the normal range (Figure 3). Pb exposure reduced the level of GPx and SOD in fish significantly (*P* < 0.05), but levels of GPx and SOD recovered in the Pb + P16 group. However, dietary supplementation of *L. reuteri* P16 alone had no significant effect on those parameters, except SOD level, which increased significantly than that in the others.

### Lysozyme and Phagocytic Activities

**Figure 4:**
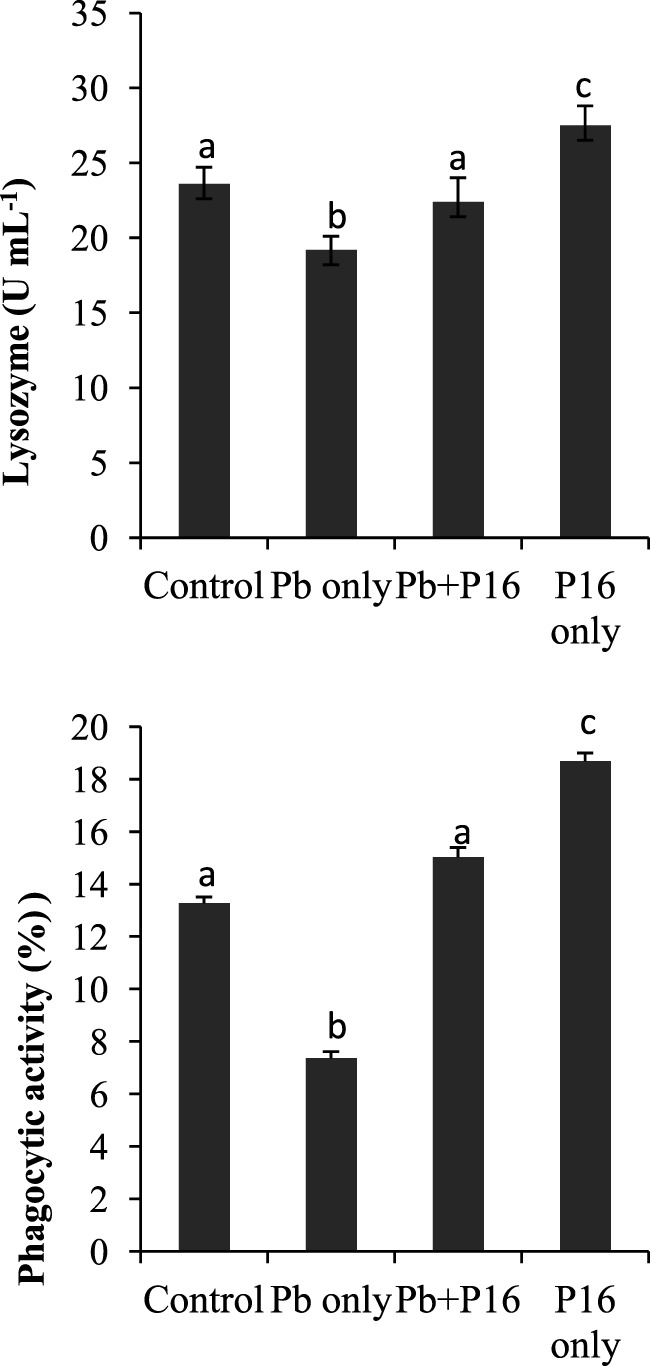
Effect of *Lactobacillus reuteri* P16 supplementation on the serum lysozyme and leukocyte phagocytoc activities of *Cyprinus carpio*. Significant differences between groups are indicated with different superscript letters. Results are presented as mean ± SEM (*n* = 30).

Pb exposure caused a profound decline in innate immune parameters, such as serum lysozyme and phagocytic activities (Figure 4). However, dietary administration of *L. reuteri* P16 helped in recovering lysozyme and phagocytic activities, as evident in the Pb + P16 group. Moreover, dietary administration of *L. reuteri* P16 only markedly increased (*P* < 0.05) lysozyme (27.5 U mL^−1^) and phagocytic activities (18.68%), when compared with those in the control or any other group (Figure 4).

### Intestinal Enzymatic Activities

**Figure 5:**
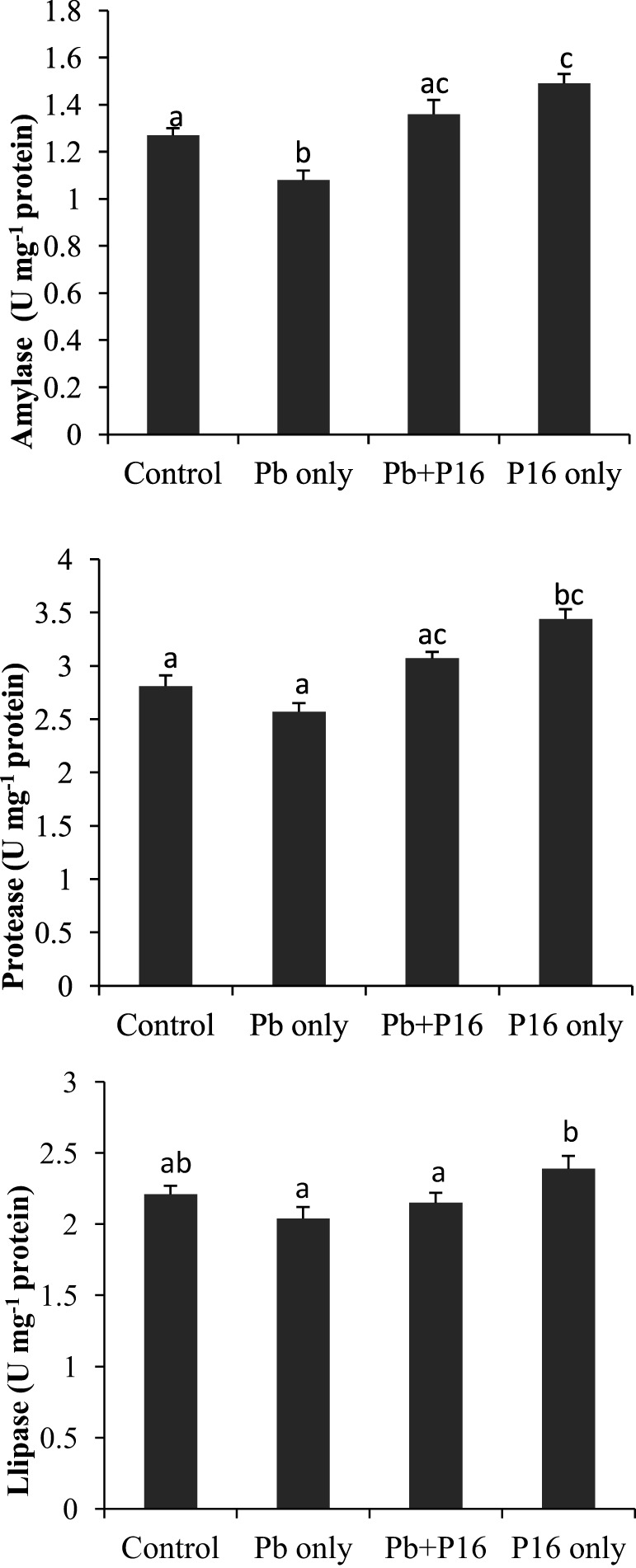
Influence of *Lactobacillus reuteri* P16 supplementation on the intestinal enzymatic activities of *Cyprinus carpio*. Significant differences between groups are indicated with different superscript letters. Results are presented as mean ± SEM (*n* = 6).

Intestinal enzymatic activities are shown in Figure 5. Activities of amylase, protease, and lipase declined in the Pb-exposed group, and the differences were significant only in case of amylase activity. The alterations of amylase and protease activities were significantly reversed in the Pb + P16 group. The lipase activity was slightly recovered in Pb + P16 group. Furthermore, dietary administration of *L. reuteri* P16 only resulted in significantly higher amylase (1.49 U mg^−1^ protein) and protease (3.43 U mg^−1^ protein) activities, when compared with that in the control group. However, *L. reuteri* P16 only had no significant effects on lipase activity.

### Quantification of Intestinal Microbiota

**Figure 6:**
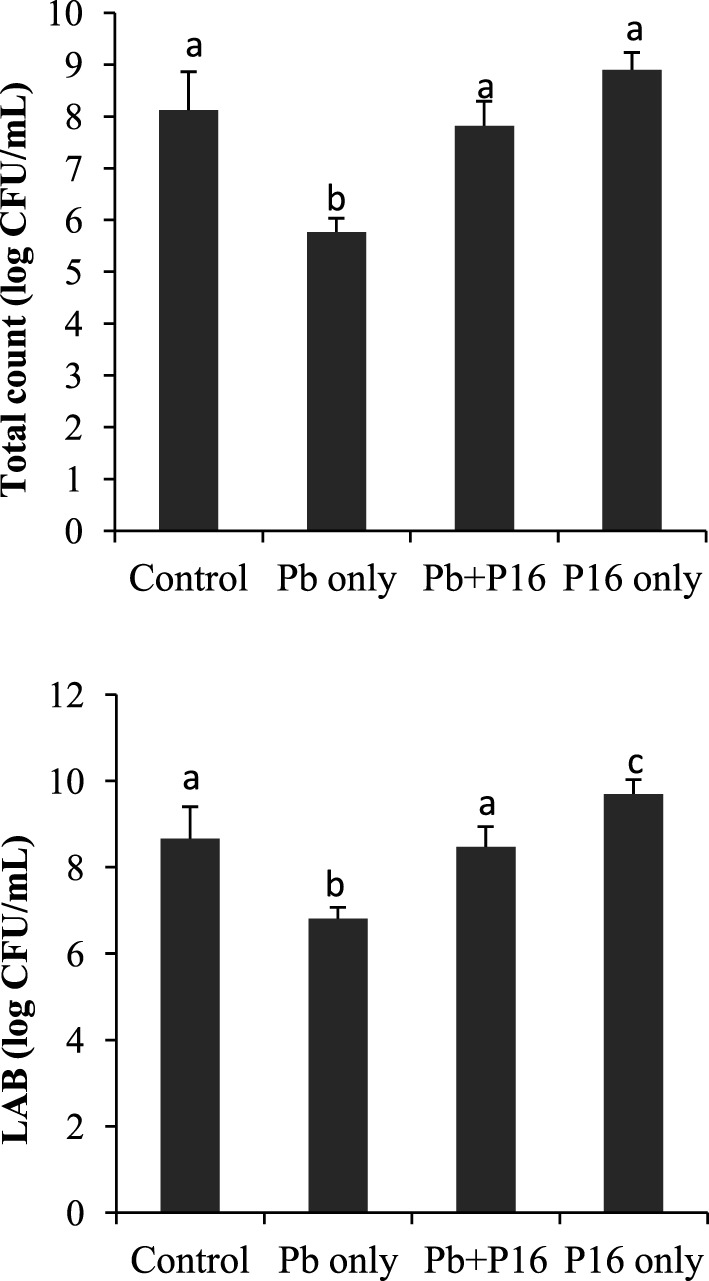
Effect of waterborne Pb exposure and dietary *Lactobacillus reuteri* P16 on gut bacterial population of *Cyprinus carpio*. The different superscript letters indicate statistically significant differences between groups. Results are expressed as mean ± SD (*n* = 6).

Pb exposure caused a profound decline in gut microbial diversity (Figure 6). Population of total bacteria and LAB counts decreased significantly in Pb-only group, as compared with that of the other groups. However, probiotic treatment had considerable effect on intestinal microbiota. Alterations of bacterial population were reversed in the Pb + P16 group. Moreover, *L. reuteri* P16 treatment increased the LAB population significantly in *L. reuteri* P16-treated group.

### Pb Levels in Tissues

Pb levels detected in various tissues of carps are shown in Table 5. Pb exposure significantly increased the level of Pb in gills (5.17 µg g^−1^), spleen (3.86 µg g^−1^), liver (8.92 µg g^−1^), kidney (26.33 µg g^−1^), and intestines (10.27 µg g^−1^), when compared with those in the control group. Supplementation with *L. reuteri* P16 significantly decreased Pb accumulation in all tissue, except the intestine, when compared with the Pb-only group. But, the level of Pb accumulation in the Pb + P16 group was higher (*P* < 0.05) than that in the control group. Furthermore, Pb accumulation in the gills, spleen, liver, kidney, and intestine of the P16 group was 0.011, 0.05, 0.19, 0.28, and 0.09 µg g^−1^, respectively, which were significantly lower than those in the Pb + P16 group, but no significant differences were observed with those in the control group.

**Table 5:** Effect of *Lactobacillus reuteri* P16 supplementation on the Pb levels in the tissues of *Cyprinus carpio*.

Group	Concentration of Pb in tissues (μg g^**−**1^ of wet tissue)				
	Gill	Spleen	Liver	Kidney	Intestine
Control	0.014 ± 0.001^a^	0.07 ± 0.01^a^	0.24 ± 0.02^a^	0.37 ± 0.08^a^	0.11 ± 0.02^a^
Pb only	5.17 ± ± 0.13^b^	3.86 ± 0.07^b^	8.92 ± 0.13^b^	26.33 ± 2.28^b^	10.27 ± 0.36^b^
Pb + P16	2.94 ± 0.14^c^	2.11 ± 0.08^c^	5.73 ± 0.26^c^	17.08 ± 1.17^c^	8.92 ± 0.61^b^
P16 only	0.011 ± 0.001^a^	0.05 ± 0.01^a^	0.19 ± 0.03^a^	0.28 ± 0.03^a^	0.09 ± 0.004^a^

*Values in the same column with different superscript small letters are significantly different (*P* < 0.05)*.

*Values are presented as mean ± SD (*n* = 30 fish in each group)*.

### Immune Gene Expression

**Figure 7:**
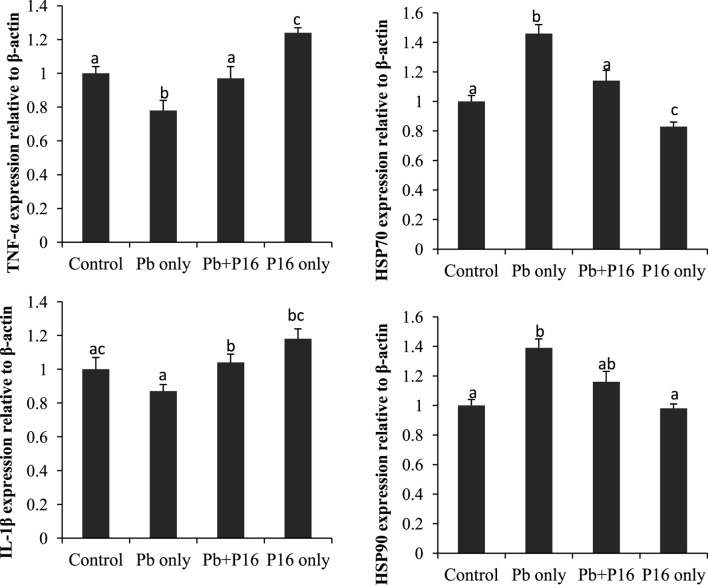
Effects of dietary administration of *Lactobacillus reuteri* P16 on the relative mRNA expression levels of TNF-α, IL-1β, HSP70, and HSP90 in the head-kidney of *Cyprinus carpio*.

Expression profiles of TNF-α, IL-1β, HSP70, and HSP90 in the head-kidney of the fishes at the end of the trial (Figure 7) showed that the expression of the pro-inflammatory cytokine TNF-α was significantly lower than that in the group exposed to Pb only, but it reversed in the Pb + P16 group. Similarly, IL-1β expression, which was lower in Pb-only group, was reversed in the Pb + P16 group. However, expression of TNF-α was significantly higher in the P16 group when compared with that in any other group.

The mRNA expression of heat shock proteins (HSP70 and HSP90) was increased drastically in Pb-only exposed group, but probiotic supplementation reversed the expression to the normal level (Figure 7). Interestingly, dietary administration of *L. reuteri* P16 only decreased the expression of both HSPs, but the differences remained significant in case of HSP70.

## Discussion

Physiological status of fishes is an important factor for determining their ability to resist pathogen attacks. Fish growth performance is affected by various external factors, such as temperature, nutrition, and toxicants. Inhibition of fish growth could be induced by physiological stress during exposure to toxic substances, because stress can reduce food consumption or assimilation (35). *L. reuteri* P16 supplementation reversed the Pb-exposure induced adverse effects on growth performance and survival of *C. carpio*. Recently, Zhai et al. (18) demonstrated that dietary supplementation with *L. plantarum* ameliorated the growth performance and prevented the death of Pb-exposed Nile tilapia (*Oreochromis niloticus*). Moreover, dietary supplementation with *L. plantarum* CCFM8610 increased the growth rate, decreased the FCR, and completely prevented the death of Cd-exposed fish (14). Also, significantly lower FCR in the P16 group reveals the fact that carp consumed dietary nutrients more efficiently when feed was supplemented with *L. reuteri*, which was consistent with numerous studies that reported significantly higher growth performances in fish fed with diet supplemented with the probiotic *Lactobacillus* (24).

Hematology has been widely used to evaluate the health status of animals exposed to environmental toxicants, and it provides information on digestive function, nutrient status, and metabolic activity of fish (36). The significant reduction in hematological parameters, such as RBC, WBC, Hct, and Hb concentration after waterborne Pb exposure was consistent with earlier reports (4). Heavy metal exposure generally induces lysis of erythrocytes in aquatic animals, leading to the depletion of Hct and Hb (37). Lead directly inhibits the synthesis of Hb by inhibiting various key enzymes involved in the heme synthesis pathway and reduces the life span of RBCs by increasing the fragility of cell membranes. The combined effect of these two processes leads to anemia (38). However, dietary *L. reuteri* P16 was effective in recovering those hematological parameters. The increased of RBC and WBC may be attributed to the effective antioxidant role of *L. reuteri* P16 (discussed below). Serum total protein, an important indicator of humoral immune system and health status of fish, was recovered in Pb + P16 group. Increase in serum protein level might be partially related with higher WBC, which is a major source of serum protein production (36). Furthermore, improved protein level in the group fed with *L. reuteri* P16 only indicated improved health status of fishes owing to probiotic supplementation.

Hepatocellular injury in fish may be attributed to stress, nutrition imbalance, diseases, or pollutants. AST and ALT levels are biological indicators of hepatic health status (39). The restoration of serum AST and ALT levels in the Pb + P16 group indicated the protective effect of *L. reuteri* P16 against Pb-induced damage of liver and heart in *C. carpio* (40). Recently, Zhai et al. (14, 18) demonstrated that dietary administration of *L. plantarum* could restore alterations of AST and ALT in Nile tilapia. Similarly, changes in ALP activity, which an indicator of environmental stress, was restored in Pb + P16 group. However, diet-borne Pb exposure had no significant effect on ALP activity in rockfish (*Sebastes schlegelii*) (4). Cholesterol is a critical structural component of membranes and a precursor of all steroid hormones. The increase in cholesterol level in the Pb exposure group may be a sensitive indicator for metal-induced environmental stress (41). The blood cholesterol level was revered by dietary *L. reuteri* P16 supplementation. A considerable increase in the cholesterol of Nile tilapia (42) and rockfish (4) exposed to Pb was reported. Furthermore, creatinine level, which is an indicator of kidney function, is normally presents in low quantities in fishes (43). However, higher levels were present in the Pb-only group. Therefore, the higher cholesterol level in blood may be due to liver and kidney damage.

Malondialdehyde, GPx, and SOD are biomarkers of heavy metal-induced oxidative stress in aquatic animals. MDA, the main component of lipid peroxides, has strong biotoxicity and can damage the structure and function of cells (44). The first line of defense against oxidative stress consists of the antioxidant enzymes SOD, CAT, and GPx, which convert superoxide radicals into hydrogen peroxide and then into water and molecular oxygen (45). SOD comprises a group of metalloenzymes that catalyzes the dismutation of superoxide to hydrogen peroxide and plays a crucial role against the toxic effects of superoxide radicals in aerobic organisms (46). In this study, higher oxidative stress in the Pb exposure group was manifested by higher (*P* < 0.05) MDA levels and lower (*P* < 0.05) GPx and SOD levels. However, *L. reuteri* P16 supplementation reduced oxidative stress in carp. This study indicates that *L. reuteri* P16 can directly mitigate Pb-induced oxidative stress. Recently, Zhai et al. (14, 18) demonstrated that dietary supplementation of *L. plantarum* could reduce waterborne Pb-induced oxidative stress in Nile tilapia. Those results, together with those of the present study, indicate that probiotic lactobacilli have protective effects against Pb-induced oxidative damage in fish. MPOs are known to play an important role in cellular defenses against various bacterial infections and can generates oxidants from H_2_O_2_ and a range of co-substrates (47). Recently, Paul et al. (8) reported that exposure to lead acetate could significantly reduce MPO levels in the macrophages of the freshwater fish *C. punctatus*. Dietary *L. reuteri* P16 supplementation was effective in recovering the MPO level in Pb-exposed fish and thereby improving host defense mechanisms.

Fish generally depend on non-specific immune responses. Lysozymes constitute the first line of defense following immune challenge to inhibit the adhesion and colonization of microorganisms (36); they split the β-1,4 glycosidic linkages between the *N*-acetyl glucosamine and *N*-acetyl muramic acid of the peptidoglycan in the bacterial cell wall, causing bacteriolysis and thereby controlling infection. By contrast, phagocytosis is responsible for early activation of the inflammatory response before antibody production and is mediated by phagocytic cells, such as neutrophils, monocytes, and macrophages, in fish (48). Significantly lower phagocytic and lysozyme activity r in the Pb-exposed group was in line with earlier reports (8, 18). But, those activities were recovered in the Pb + *L. reuteri* P16 supplementation group. Several studies have demonstrated that probiotic feeding could significantly improve various innate immune parameters in fish (22, 24, 25).

Another possible explanation for the stimulation of growth by potential probiotic *L. reuteri* P16 may be related to the induction of the expression of digestive enzymes in carp intestines by dietary P16, which boosts the natural digestive enzyme activity, and thereby the growth performance, of the host (49). This increased digestive enzymatic activity might be a means of protection against the adverse effects of Pb on fish. Very recently, Zhai et al. (18) reported that dietary administration of probiotic *L. plantarum* increased amylase and protease activity in Nile tilapia exposed to waterborne Pb. Better feed consumption (as revealed by reduced FCR) and digestion in *L. reuteri* P16 supplemented groups may be related to increased digestive enzyme activity and thereby increased appetite in fish. The gut microbiota is in direct contact with the intestinal mucosa constantly and plays a vital role in maintaining fish health (50). Pb exposure decreased LAB counts and total gut microbial populations. Pb-induced alterations in the gut microbiota may cause dysbiosis in carps, which can be related to the adverse effects on growth performance, antioxidant defense system, and hematological parameters of Pb-exposed fish. However, dietary supplementation with *L. reuteri* P16 moderately restored the gut dysbiosis. *L. reuteri* P16 supplementation increased LAB population in Pb-exposed fish. Alleviation of heavy metal-induced toxicities in fish through dietary administration of lactobacilli strains has been reported (13, 14, 18). Moreover, increased population of LAB may enhance Pb sequestration in gut, owing to its cellular accumulation and bioremoval.

Tissue-specific accumulation can be a sensitive indicator of metal exposure in aquatic toxicology. Exposure to waterborne Pb causes significant accumulation of Pb in various tissues of carp, with highest accumulation in kidney. Exposure of *S. schlegelii* to dietary Pb revealed that the highest Pb accumulation took place in the kidney (8). Similarly, varying Pb accumulation was recorded in tissues of Nile tilapia exposed to waterborne Pb (18). Differences in the extent of metal accumulation between tissues are considerably related to ecological needs and metabolic activity (51). The uptake of toxic heavy metals in aquatic animals generally occurs *via* two major routes: waterborne and diet borne. Waterborne Pb can be taken up by gills of fish, and it may contaminate food and be absorbed *via* intestines, then accumulate in tissues including the kidneys and liver (52). *L. reuteri* P16 used in this study has been reported to bind effectively to Pb *in vitro* (28). Therefore, intestinal *L. reuteri* P16 could bind secreted Pb before re-absorption from intestines, which increases Pb excretion through the feces of fish and inhibits intestinal absorption (22). This may a reason for the reduction in Pb levels in the Pb + *L. reuteri* P16 group.

The inflammatory response is a key element in the innate immune response system and is primarily mediated by cytokines (53). Very little is known about the immunotoxicology of heavy metals at the genetic level in fish. The cytokines IL-1β and TNF-α are primarily produced by monocytes and macrophages and regulate multiple aspects of the immune response. TNF-α affects tissue vasculature in inflammation and also induces acute phase proteins from the liver (54). The observed downregulation of TNF-α and IL-1β implicates NF-κB signaling pathway. However, *L. reuteri* P16 supplementation had a positive on TNF-α and IL-1β expression. In a previous study, Paul et al. (8) recorded decreased TNF-α level in sera and cell lysates of freshwater fish *C. punctatus* exposed to lead acetate. Based on the results of this study, it can be stated that Pb renders the fish in an immunocompromised and inflammatory state. Thus, TNF-α and IL-1β are downregulated, probably by the action of the heavy metal (8), but probiotic supplementation reversed these effects on the expression of those genes.

The synthesis of heat shock proteins (Hsps) increases in response to various physical and chemical stressors, including temperature and metal stress, and consequently these proteins can be good environmental stress biomarkers (55). Expression of HSPs which increased in Pb-exposure group, was further decreased in the *L. reuteri* P16 supplementation group and thus alleviated stress condition in fish. In agreement with the results of our study, Mohapatra et al. (19) demonstrated that dietary supplementation of a probiotic mixture consisting of *Bacillus subtilis, Lactococcus lactis*, and *Saccharomyces cerevisiae* decreased the expression of HSP70 in *Labeo rohita*. Stress-reducing factors produced by probiotics might have lowered the HSP levels in fish, reduced Pb-induced stress, and resulted in better growth and immunity.

**Figure 8:**
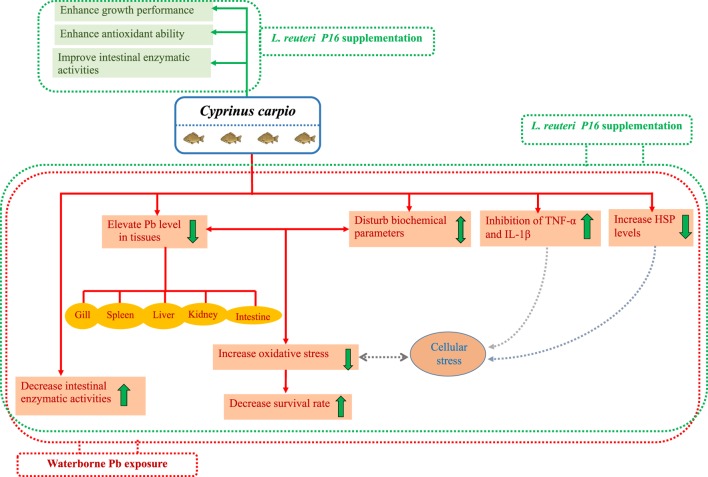
Potential beneficial effects and protective mechanism of probiotics against Pb toxicity in *Cyprinus carpio*.

Interestingly, co-treatment with Pb and *L. reuteri* P16 reversed Pb-induced adverse effects on the carp species studied. The potential beneficial effects and protection by probiotics against Pb toxicity in carp has been shown in Figure 8. The hematological parameters indicated the ability of *L. reuteri* P16 to stimulate erythropoiesis, thereby increasing the oxygen transport, and a similar result had been reported earlier (13). Previously, we have demonstrated the excellent Pb-binding ability of this strain (28). Therefore, supplementing diets with this strain can reduce intestinal absorption of Pb and reduce Pb accumulation in other tissues. The strain *L. reuteri* P16 possess strong antioxidant abilities (28). In this study, co-treatment with Pb and *L. reuteri* P16 reduced production of MDA and increased the activities of antioxidant enzymes (SOD and GPx). Thus, *L. reuteri* P16 alleviates the Pb-induced oxidative stress in carps. However, the underlying role of antioxidative activity of this strain against Pb-induced oxidative stress and dysfunction of the gut barrier need to be investigated further. In addition, co-treatment with Pb and *L. reuteri* P16 reversed the expressions of pro-inflammatory cytokines (TNF-α and IL-1β), which are associated with the NF-κB signaling pathway and subsequent decreases in reactive oxygen species production and prevents Pb-induced cellular apoptosis. However, comprehensive Pb tolerance mechanisms in LAB strains have not been investigated. Therefore, Pb stress response network in LAB strains are yet to be elucidated. Furthermore, key proteins and pathways involved in Pb tolerance of lactobacilli strains are unknown. Hence, isobaric tags for relative and absolute quantification (iTRAQ)-based comparative and functional proteomic approaches could be utilized to explore Pb-tolerance related to key proteins and pathways in *L. reuteri* strains. Moreover, at this early stage of research, we do not know how probiotics repair the metabolic damages occurring in fish exposed to heavy metals; therefore, efforts should be made in the future to incorporate multiple targets (genes, proteins, and metabolites) for exploring heavy metal defense mechanisms of probiotics in hosts.

Notably, treatment with *L. reuteri* P16 alone did not exert any adverse effects on growth performance, hematological and blood biochemical parameters, intestinal enzymatic activities, and intestinal microbiota. In addition, growth rate and feed utilization was greater in the group fed with only *L. reuteri* P16. This finding is in agreement with that of several earlier reports, which demonstrated that probiotics could boost the growth performance of fish. Furthermore, this strain exhibited higher Pb-removing antioxidant potential *in vitro* (28). Results of the present study suggest that *L. reuteri* P16 supplementation is safe for the carp species studied.

## Conclusion

Lead has deleterious effect on physiological and immune functions in fish. Exposure of fish to sublethal concentrations resulted in significant Pb accumulation in specific tissues, whereas *L. reuteri* P16 supplementation strongly reduced this. This strain significantly increased the feed utilization, growth performance, and survival of Pb-exposed fish. *L. reuteri* P16 supplementations recovered Pb toxicity related biochemical parameters, alleviated oxidative stress, and re-established gut microbial population and intestinal enzymatic activities. Furthermore, *L. reuteri* P16 supplementation was effective in reducing the Pb-exposure-induced expression of heat shock proteins. Overall, *L. reuteri* P16 supplementation showed considerable effectiveness in attenuating the changes caused by Pb-induced toxicity. Furthermore, administration of *L. reuteri* P16 by itself did not exert any adverse effects on fish health, suggesting that the dietary supplementation of this strain is sage. Results of the study indicate that *L. reuteri* P16 can cope with heavy metal stress owing to induction of cellular defense and repair system, but the underlying mechanisms of Pb tolerance of this strain yet to be explored. Therefore, these results suggest that *L. reuteri* P16 has potency as a novel dietary supplement to prevent safety problems induced by lead pollution in carp aquaculture. Therefore, *Lactobacillus* strains with both good Pb-binding and antioxidative capacities, such as *L. reuteri* P16, can be considered for utilization as dietary supplement for the prevention of Pb contamination aquaculture.

## Author Contributions

SG, VS, and SP designed the work. SG contributed significantly in lab works. SY helped him in conducting the experiments. SGK SWK, JK, and SH helped in sample collection and immunological studies. SG wrote the manuscript. All authors reviewed and contributed significantly to improve the manuscript. JJ contributed to study the immune gene expression analysis. JJ, SY, and HK contributed to statistical analysis of the data.

## Conflict of Interest Statement

The authors declare that the research was conducted in the absence of any commercial or financial relationships that could be construed as a potential conflict of interest.
