# Corrigendum: Therapeutic Effect of Intestinal Autochthonous *Lactobacillus reuteri* P16 Against Waterborne Lead Toxicity in *Cyprinus carpio*

**DOI:** 10.3389/fimmu.2018.02208

**Published:** 2018-09-26

**Authors:** Sib Sankar Giri, Saekil Yun, Jin Woo Jun, Hyoun Joong Kim, Sang Guen Kim, Jeong Woo Kang, Sang Wha Kim, Se Jin Han, V. Sukumaran, Se Chang Park

**Affiliations:** ^1^Laboratory of Aquatic Biomedicine, College of Veterinary Medicine, Research Institute for Veterinary Science, Seoul National University, Seoul, South Korea; ^2^Department of Biotechnology, Periyar Maniammai University, Thanjavur, India

**Keywords:** probiotics, lactic acid bacteria, Pb, common carp, oxidative stress, immune parameters, gene expression

There is an error in the Funding statement. The correct number for the Cooperative Research Program for Agriculture Science and Technology Development (Supportive managing project of Center for Companion Animals Research) by Rural Development Administration is PJ013985032018.

The authors apologize for this error and state that this does not change the scientific conclusions of the article in any way.

## Conflict of interest statement

The authors declare that the research was conducted in the absence of any commercial or financial relationships that could be construed as a potential conflict of interest.

