# Supplementary material for: Therapeutic Effect of Intestinal Autochthonous Lactobacillus reuteri P16 Against Waterborne Lead Toxicity in Cyprinus carpio
Source: Front Immunol. 2018 Aug 7;9:1824. doi: 10.3389/fimmu.2018.01824 (PMC6090060; doi:10.3389/fimmu.2018.01824)
Supplement: Supplementary file 1 [file table_1.docx]

**SUPPLEMENTARY TABLE**

**Importance of parameters studied**

| **SL. No.** | **Parameters studied** | **Interest of determination** |
| --- | --- | --- |
| 1 | Hematological parameters   - WBC - RBC - Hb - Serum protein - Cholesterol | Hematological parameters indicate the health status of fish  WBCs play vital role in innate immunity of fish during inflammation.  RBCs are principle in delivering oxygen to tissues. The transport of O_2_ and CO_2_ within the blood are intricately related to the electrolytes and acid-base status of the RBCs.  Hb in oxygen-binding protein in blood. It transport oxygen from gas-exchange organs to peripheral tissues.  Serum protein level indicator of humoral immune system.  Cholesterol is fatty substance that does not mix with blood. It mains membrane structural integrity and fluidity but too high amount of cholesterol can form fatty deposits in arteries. |
| 2 | Blood Biochemical Parameters   - MDA - MPO - AST - ALT - ALP - SOD - GPx - Creatinine - Lysozyme - Leucocyte phagocytic activity | MDA is an indicator of lipid peroxidation process.  MPO is very abundant heme-proteins found in different leucocyte populations of invertebrate and vertebrate organisms. MPO activity is assumed to be good markers of leucocyte activation  AST and ALT levels are indicator of hepatic health function. Higher AST/ ALT levels indicates hepatic damage.  ALP an enzyme that catalyses the dephosphorylation of a range of molecules such as nucleotides and proteins.  SOD is a antioxidant enzyme that play a key role in the defense against ROS by transforming superoxide anions into hydrogen peroxide, which is detoxified by both GPx and CAT activities  Elevated creatinine level signifies impaired kidney function or kidney disease.  The lysozyme is a primary marker of fish defense system, which causes of lysis of pathogens, and activation of the complement system and phagocytes through opsonin.  Activation of phagocytes can generate superoxides, hydrogen peroxide, and hydroxyl radical during the period of intense oxygen consumption called respiratory burst. |
| 3 | Intestinal Enzymatic activities   - Amylase - Protease - Lipase | Level of digestive enzymes may be used as a comparative indicator of food acceptance, and digestive capacity of fish. |
| 4 | Quantification of Intestinal microbiota | The intestinal microbiota of fish plays a key role in nutritional function, enhances growth performance as well as stimulation of the  host immune system and resistant against pathogens |
| 5 | Immune gene expression in head-kidney   - IL-1β - TNF-α - HSP70 - HSP90 | The cytokines IL-1β and TNF-α are primarily produced by monocytes and macrophages and regulate multiple aspects of the immune response.  HSPs function as helper molecules or chaperones for all protein and lipid metabolic activities of the cell, and it is recognized that the up-regulation in response to stress is universal to all cells and not restricted to heat stress |
